# The effect of 5-aminolevulinic acid on cytochrome *c *oxidase activity in mouse liver

**DOI:** 10.1186/1756-0500-4-66

**Published:** 2011-03-17

**Authors:** Shun-ichiro Ogura, Kouji Maruyama, Yuichiro Hagiya, Yuta Sugiyama, Kyoko Tsuchiya, Kiwamu Takahashi, Fuminori Abe, Kenji Tabata, Ichiro Okura, Motowo Nakajima, Tohru Tanaka

**Affiliations:** 1 Endowed Research Section (ALA), Frontier Research Center, Tokyo Institute of Technology, Yokohama, Japan; 2 Shizuoka Cancer Center Research Institute, Shizuoka, Japan; 3 SBI ALApromo Co., Ltd., Tokyo, Japan

## Abstract

**Background:**

5-Aminolevulinic acid (ALA) is a precursor of heme that is fundamentally important in aerobic energy metabolism. Among the enzymes involved in aerobic energy metabolism, cytochrome *c *oxidase (COX) is crucial. In this study, the effect of ALA on cytochrome *c *oxidase activity was measured.

**Findings:**

c57BL/6N species of mice were administered ALA orally for 15 weeks. After ALA administration, mice were sacrificed and livers were obtained. COX activity in mitochondria from ALA-administered mouse livers was 1.5-fold higher than that in mitochondria from PBS-administered mouse livers (P < 0.05). Furthermore, ATP levels in ALA-administered mouse livers were much higher than those in PBS-administered mouse livers. These data suggest that oral administration of ALA promotes aerobic energy metabolism, especially COX activity.

**Conclusions:**

This is the first report of a drug that functions in aerobic energy metabolism directly. Since COX activity is decreased in various diseases and aging, the pharmacological effects of ALA will be expanding.

## Backgrounds

The activity of cytochrome *c *oxidase (complex IV, COX, EC 1.9.3.1) in the electron transfer chain is fundamentally important in aerobic energy metabolism. This enzyme catalyzes electron transfer from cytochrome *c *to molecular oxygen, reducing the latter to water, and yields substantial energy that drives the formation of a proton gradient that is then employed to synthesize ATP [1]. The activity of COX is also important in aging and several diseases. Age-related decreases in COX activity have also been reported in many species (e.g., *Drosophila melanogaster *[2], housefly [3], rodents [4,5]). Müller-Höcker observed that COX-negative muscle fibers were increased with age in humans and animals [6-8], showing that the improvement of COX activity is important for anti-aging. COX activity has been shown to be diminished in mitochondria from the brains of Alzheimer's disease patients [9,10]. In human cancer cells, p53 regulates COX activity through *synthesis of cytochrome c oxidase 2 *(SCO2) [11], causing a metabolic change called the Warburg effect [12]. COX activity is also inhibited by nitric oxide (NO), which is a signaling molecule involved in many pathophysiological processes (e.g., smooth muscle relaxation, inflammation, neurotransmission, apoptosis) [13]. COX inhibition is also observed in diabetes. COX levels in sensory neurons from diabetic rats was downregulated by 29% compared to that from normal rats [14], and advanced glycation end products, which are implicated in diabetic complications, inhibit COX activity by inducing the expression of inducible nitric oxide synthase (iNOS) [15]. Therefore, COX activity is important, and to our knowledge there is no drug that activates COX.

5-Aminolevulinic acid (ALA) is widely distributed in both plant and animal cells, and it is the common precursor of tetrapyrrole compounds. In animal cells, ALA is formed from glycine and succinyl CoA by ALA synthase in mitochondria. Administration of ALA enhances the cancer-specific accumulation of porphyrins. ALA-induced porphyrins can be fluoresced and visualized in neoplastic regions. This technique exploits ALA-induced differences in fluorescent signatures between normal and cancer tissues. Therefore, the technique is generally termed fluorescence diagnosis or photodynamic diagnosis [16].

On the other hand, in normal tissue, iron ion is inserted into porphyrin to form heme in mitochondria, which is incorporated into hemoproteins and cytochromes. Because COX and its substrate (cytochrome *c*) are major hemoproteins, heme is fundamentally important to COX activity. In this study, to increase COX activity, ALA was administered to mice, and COX activity in the liver was measured.

## Materials and methods

### Materials

5-Aminolevulinic acid hydrochloride was purchased from Cosmo Bio Co., Ltd. (Tokyo, Japan). Mitochondria isolation and cytochrome *c *oxidase assay kits were purchased from Sigma Aldrich (St. Louis, MO, USA), and an ATP assay kit for animal tissues (TA100, Toyo B Net, Tokyo, Japan) was purchased from Wako Pure Chemical Industries Ltd. (Osaka, Japan)

### Animals and in vivo experiments

c57BL/6N mice (female, 6 weeks old) were purchased from CREA Japan (Tokyo, Japan). All mice were housed and used for experiments in accordance with standard ethical guidelines for the care and use of laboratory animals [Science Council of Japan; *Guidelines for Proper Conduct of Animal Experiments *(2006)], and this study was approved by the Animal Experiment Ethics Committee of Shizuoka Cancer Center.

ALA was administered orally for 15 weeks. As a stock solution, 4 mg/ml ALA in PBS was prepared, and the ALA stock solution was added to create a 1:100 dilution (final concentration: 0.04 mg/ml, approximately 10 mg/kg/day). PBS was added to drinking water for the control group.

### Measurement of COX activity in liver mitochondria

Mouse livers were extracted, and liver mitochondria were obtained using a mitochondria isolation kit (Sigma-Aldrich). In brief, fresh livers were washed and homogenized (rotor-stator homogenizer (T10 basic Ultra-Turrax^®^, IKA, Staufen, Germany) at 15,000 rpm) in extraction reagent. The homogenate was centrifuged at 600 ×*g *for 5 min, and then the supernatant was further centrifuged at 11,000 × *g *for 10 min. The pellet was suspended in storage buffer and used as a mitochondrial fraction. Protein concentrations were determined by the Bradford assay (Bio-Rad Laboratories, CA). COX activity in liver mitochondria was measured using a cytochrome *c *oxidase assay kit. In brief, 5 μg of the mitochondrial fraction were diluted with enzyme dilution buffer containing 1 mM *n*-dodecyl β-d-maltoside. Ferrocytochrome *c *(reduced cytochrome *c *with dithiothreitol) was then added to the sample, and COX activity was measured by the decrease in absorption at 550 nm. The difference in extinction coefficients between reduced and oxidized cytochrome *c *is 21.84 at 550 nm [17]. One unit of COX activity was defined as the oxidization of 1.0 μmole of ferrocytochrome *c *per min at pH 7.0 at 25°C.

### Western blot analysis

Western blot analysis was performed as previously described with some modifications [18]. Liver mitochondria, obtained as described above, were separated by 15% SDS-PAGE and then transferred onto polyvinylidene fluoride membranes (Millipore, Bedford, MA). After incubating in 5% nonfat dry milk in TBST (25 mM Tris, pH 7.5, 150 mM NaCl, and 0.1% Tween 20) at room temperature for 1 h, the membranes were incubated for 1 h with mouse anti-COX IV-1 antibody (1:200 dilution in TBST with 5% nonfat dry milk, Santa Cruz Biotechnology, Santa Cruz, CA) followed by incubation in a secondary anti-mouse IgG coupled to horseradish peroxidase (1:3000 dilution in TBST, Cell Signaling, Danvers, MA). Target proteins were detected using an ECL kit (GE Healthcare, UK), and the image was captured using an LAS-4000 imager (GE Healthcare).

### Measurement of ATP level in liver

For measuring ATP, cellular ATP in livers was extracted using an ATP extraction kit of an ATP assay kit (TA100, Toyo B Net). In brief, small pieces of livers (about 0.1 g) were washed once with PBS, resuspended in ATP extraction reagent, and then centrifuged at 1,000 ×*g *for 10 min. The supernatant was used for the ATP assay. The ATP level was quantitatively measured using an ATP assay kit with luciferin and luciferase according to the protocol provided by the manufacturer (Toyo B Net).

## Results and discussion

**Figure 1:**
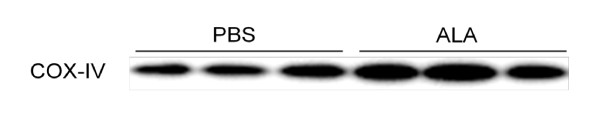
**Western blot analysis of the mouse liver mitochondrial fraction**. Ten micrograms of liver mitochondria, obtained as described in the Material and Methods section, were separated by 15% SDS-PAGE and then electrotransferred on to a PVDF membrane and Western blot analysis were performed using COX IV-1 antibody. The average COX IV expression level in the ALA administered group was 1.6-fold higher than that of the control group.

No mice died due to ALA administration for 15 weeks, and significant body weight changes were not observed, indicating that ALA has no toxicity in this condition. Western blot analysis of the mouse liver mitochondrial fraction was performed (Figure 1). COX consists of 10 nuclear-coded subunits and 3 catalytic mitochondrial-coded subunits [19]. Among these 13 subunits, subunit IV specifically binds ATP, playing an important role in COX activity [20]. Thus the antibody raised against COX IV was employed for western blot analysis in our study (Figure 1). Apparent increases in COX protein levels were observed in ALA-administered mouse livers. Approximately 1.6-fold increase was observed by densitometric scanning compared to the control.

**Figure 2:**
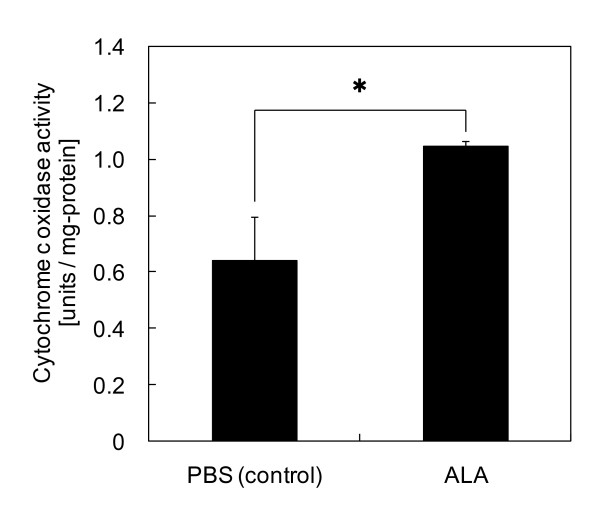
**COX activity in mitochondria from mouse livers**. COX activity was defined as the oxidization of ferrocytochrome *c*. Bars represent SD of the means of three replicate experiments. *P < 0.05 by Student's t-test.

Figure 2 illustrates COX activity in mitochondria from mouse livers. COX activity was defined as the oxidization of ferrocytochrome *c*. The average COX activity in mitochondria from ALA-administered mouse livers was 1.5-fold higher than that in mitochondria from control mouse livers, which was statistically different (P < 0.05 by Student's t-test). This finding is in good agreement with the increase in COX protein levels, as shown in Figure 1. COX activity in *Drosophila melanogaster *declined progressively with age by 33% [2], indicating that the 1.5-fold increase in activity observed in this study is substantial. Our results clearly demonstrate that both COX protein levels and activity were increased by ALA administration. This is the first report of hepatic COX activation achieved by oral administration of a natural compound.

**Figure 3:**
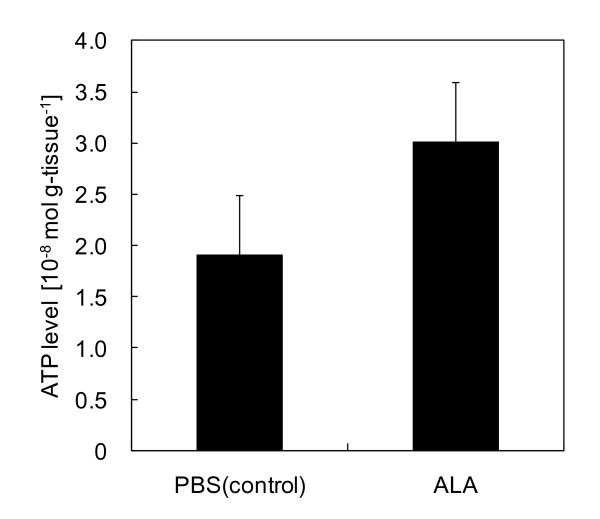
**ATP level in mouse liver lysates**. ATP was extracted and quantified using luciferin and luciferase. Bars represent SD of the means of three replicate experiments. P = 0.069 by Student's t-test.

Figure 3 shows ATP levels in mouse liver lysates. ATP was extracted and quantified using luciferin and luciferase. ATP levels in ALA administered mouse livers were 1.6-fold higher than those in control mouse livers. This apparent increase in ATP levels may be caused by COX activation in the mitochondrial electron transfer chain.

COX and cytochrome *c *are hemoproteins, and COX activity was increased by ALA administration. ALA is the precursor of protoporphyrin, and heme is produced by the insertion of iron. Therefore, ALA administration can result in heme production in the mouse liver, and it is possible that COX activity was increased by ALA administration. Heme deficiency selectively decreased the activity and protein content of COX by 95% [21], that is observed in aging. This phenomenon strongly suggests the amount of heme is important for the COX activity. In addition, COX requires some cofactors, namely iron and copper ions. In this study, iron and copper were not administrated directly. However, these elements were easily obtained by the animals via routine feeding. There is a possibility that these elements have key roles in the maintenance of COX following administration of ALA. Because we detected increases in COX activity and protein levels, further analysis may be needed for clarification. In addition to COX, Complex II and Complex III are also hemoproteins. These proteins may also be upregulated by ALA administration.

Coenzyme Q_10 _(CoQ_10_) is also a well-known cofactor in the aerobic respiratory electron transfer and is widely used as a nutritional supplement. Orally administrated CoQ_10 _can be taken up by mitochondria, but only to a limited extent [22,23]. On the other hand, protoporphyrin and heme are generated in mitochondria after ALA administration. This active targeting is important in ALA-mediated COX activation.

ALA is used in various fields, especially cancer diagnosis and therapy. There is no report of toxic side effects caused by ALA administration. In the present study, no toxicity was observed during long-term ALA administration, indicating its safety. Moreover, ALA supplementation has been used in several fields to date. Wang et al. demonstrated that ALA and vitamin C supplementation improved iron status including hemoglobin levels in sows and laying hens [24,25]. Yamazaki et al. revealed that ALA and iron ion administration caused a stimulatory effect on hair growth [26]. These findings strongly suggest that ALA is a potentially useful nutritional supplement.

In conclusion, we demonstrated improvement of COX activity by ALA administration, suggesting the further potential benefits of ALA. If ALA activates COX activity, ALA would be a promising candidate of treatment option for aging, diabetes, Alzheimer's disease, chronic inflammation, and cancers.

## Competing interests

The authors declare that they have no competing interests.

## Authors' contributions

SO designed the study, established and perfomeed cytochrome *c *oxidase assay, perfoemed data analysis, wrote and revised the final manuscript. KM, YH, YS, KTsu, KTaka established mouse liver homogenate, perfomed cytochrome *c *oxidase assay and ATP assay. FA, KTaba, IO, MN, TT provided direction and oversight of the experiments, and helped revise the final manuscript. All authors read and approved the final manuscript.
